# Disrupted prevention: condom and contraception access and use among young adults during the initial months of the COVID-19 pandemic. An online survey

**DOI:** 10.1136/bmjsrh-2020-200975

**Published:** 2021-03-11

**Authors:** Ruth Lewis, Carolyn Blake, Michal Shimonovich, Nicky Coia, Johann Duffy, Yvonne Kerr, Jill Wilson, Cynthia Ann Graham, Kirstin R Mitchell

**Affiliations:** 1 MRC/CSO Social and Public Health Sciences Unit, University of Glasgow, Glasgow, UK; 2 Sandyford Sexual Health Service, NHS Greater Glasgow and Clyde, Glasgow, UK; 3 Health Improvement Department, NHS Lanarkshire, Bothwell, South Lanarkshire, UK; 4 Public Health, NHS Lothian, Edinburgh, UK; 5 Department of Psychology, University of Southampton, Southampton, UK

**Keywords:** contraception behavior, sexually transmitted diseases, COVID-19, sexual health, reproductive health, health services accessibility

## Abstract

**Background:**

The initial response to COVID-19 in the UK involved a rapid contraction of face-to-face sexual and reproductive health (SRH) services and widespread use of remote workarounds. This study sought to illuminate young people’s experiences of accessing and using condoms and contraception in the early months of the pandemic.

**Methods:**

We analysed data, including open-text responses, from an online survey conducted in June–July 2020 with a convenience sample of 2005 16–24-year-olds living in Scotland.

**Results:**

Among those who used condoms and contraception, one quarter reported that COVID-19 mitigation measures had made a difference to their access or use. Open-text responses revealed a landscape of *disrupted prevention*, including changes to sexual risk-taking and preventive practices, unwanted contraceptive pathways, unmet need for sexually transmitted infection (STI) testing, and switches from freely provided to commercially sold condoms and contraception. Pandemic-related barriers to accessing free condoms and contraception included: (1) uncertainty about the legitimacy of accessing SRH care and self-censorship of need; (2) confusion about differences between SRH care and advice received from healthcare professionals during the pandemic compared with routine practice; and (3) exacerbation of existing access barriers, alongside reduced social support and resources to navigate SRH care.

**Conclusions:**

Emerging barriers to STI and pregnancy prevention within the context of COVID-19 have the potential to undermine positive SRH practices, and widen inequalities, among young people. As SRH services are restored amid evolving pandemic restrictions, messaging to support navigation of condom and contraception services should be co-created with young people.

**Key messages**

- COVID-19-related changes to sexual and reproductive health (SRH) services and routine advice inhibited young people’s access to free condoms and contraception during the early stages of the pandemic.
- Young people reported feeling uncertain about the legitimacy of their SRH needs, confused by changes in professional advice on preventive practices, and hesitant about remote consultations.
- Restoration of SRH services should be accompanied by clear public messaging, co-produced with young people, to address these uncertainties and concerns.

## Introduction

On 11 March 2020, the World Health Organization (WHO) declared COVID-19 a pandemic. The initial response of the sexual and reproductive health (SRH) sector involved a rapid contraction of services globally, including those supporting contraception care and sexually transmitted infection (STI) prevention.^1^ In the UK, some SRH services were largely halted (eg, removal and reinsertion of long-acting reversible contraception (LARC), drop-in services for young people, asymptomatic STI testing), while others continued at limited capacity (eg, renewal of contraceptive prescriptions, distribution of free condoms), exacerbated by redeployment of some specialist SRH staff.^2 3^ At the time of writing, SRH services are still operating at reduced capacity. Access to contraceptive information and services is a right,^4^ and continuity of contraception provision during COVID-19 is recognised as essential by professional bodies in the UK^5^ and internationally.^6–9^ A range of remote workarounds employed to enable contraceptive access during the pandemic include: prescription of methods that do not require face-to-face appointments (eg, progestogen-only pill, self-administered injection); contraceptive prescriptions being sent directly to pharmacies and homes after telephone or video calls; remote repeat prescribing of the combined pill with relaxation of requirements for blood pressure and body mass index (BMI) measurement; and off-label extended use of some forms of LARC (eg, implant).^5^ Changes to service configuration, including wider use of remote tools, could provide potential opportunities (eg, for cost savings, removal of barriers to care for some), but also risk creating new inequalities, for example, by digital literacy and exclusion.^10^ Data are therefore needed regarding experiences of these changes to routine service provision and advice among those with SRH care needs.

Reduced access to SRH services has particular implications for young people – a group who already experience high rates of STIs and unplanned conceptions.^11 12^ Commentaries have highlighted specific challenges to SRH potentially facing young people during the COVID-19 pandemic,^13–15^ but there is currently little empirical evidence of their experiences of navigating access to condoms and contraception. Understanding the impacts on young people – both short- and longer-term – will be key for guiding decision-makers tasked with planning both current and future SRH provision in the context of an uncertain social and economic landscape.

This article presents data on self-reported experiences and concerns relating to condom and contraception access and use among 16–24-year-olds in Scotland during the early stages of the COVID-19 pandemic. Within Scotland, contraception and condoms are available to young people free of charge via local service models across all health boards. Our primary goal is not to quantify the scale of disruption to these services, but rather to illuminate the range of implications for young people with SRH needs, including those who do not access clinical SRH services.

## Methods

### Design and setting

Data were analysed from an online convenience sample survey of 2005 16–24-year-olds living in Scotland. The survey was conducted as part of CONUNDRUM (CONdom and CONtraception UNDerstandings: Researching Uptake & Motivations) – a mixed methods study using focus groups and an online survey to explore the multilevel factors shaping use and non-use of condoms and contraception among young people in Scotland.^16^ The study was commissioned in 2019 by three Scottish National Health Service (NHS) health boards, in partnership with Scottish Government, in response to observed declines in use of free condom schemes among 16–24-year-olds and uptake of some forms of LARC (eg, implant) among those aged under 20 years.^17^

The survey was open between 22 June and 31 July 2020 – a 6-week period immediately following the end of the first UK-wide lockdown, characterised by relaxation of some of the most severe restrictions but continued disruption to health services and daily life. Respondents were recruited via: (1) dissemination of an advertisement through stakeholder networks (eg, NHS and third-sector organisations) and (2) subsequent targeted social media advertising via YouTube, Facebook and Instagram, with the focus on increasing respondents from groups underrepresented in the sample (young men, and those living in areas of higher social deprivation). Ethics approval was granted by the University of Glasgow Medicine and Veterinary Life Sciences Ethics Committee.

### Patient and public involvement

Patient and public involvement (PPI) was prioritised and embedded throughout the CONUNDRUM study via multiple workshops and virtual meetings with over 100 sexual health stakeholders, including 60 young people, to co-develop research objectives, methods, and policy recommendations. Later-stage co-design of the survey occurred during the first UK-wide lockdown, presenting the opportunity to assess impacts of pandemic mitigation measures on young people’s access to, and use of, condoms and contraception. These specific questions were designed and piloted with input from nine young people.

### Measures

COVID-19-related change was measured by asking: “Have social distancing measures (ie, because of coronavirus) made any difference to the way you get or use condoms or contraception?” (options: I do not use condoms or contraception, No, Not sure, Yes). For those who responded Yes, a follow-up question (open-text response) asked: “Please tell us about how it has been different for you. This helps us understand how social distancing measures affect young people’s condom and contraception use”.

Analyses are presented by three categories of gender identity: women, including trans women; men, including trans men; and a derived category titled “identify gender in another way”, which includes participants identifying as non-binary, preferring to self-describe, or preferring not to say. Illustrative quotes include participants’ sexual identity, with respondents able to select (one or more) from: Asexual, Bisexual, Gay or lesbian, Heterosexual/straight, Pansexual, Queer, I prefer to self-describe (with open-text box), I prefer not to say.

### Analysis

Survey data were analysed in Stata 15. All survey respondents (n=2005) were asked about the impact of COVID-19 on their access and use of condoms and contraception. Among those who responded to this question (n=1992), 20% reported not using condoms or contraception and were excluded from subsequent analysis. This left an analytic sample of 1588 for descriptive analysis.

Qualitative analysis of open-text responses was informed by the Framework Method.^18^ An initial coding framework was developed via open coding, with data then indexed and thematically charted using a matrix. Mapping and development of themes was refined and reviewed through team-based discussion. Illustrative quotations are presented with details of participant age, gender and sexual identity.

## Results

Sociodemographic characteristics of survey respondents using condoms and/or contraception (n=1588) are presented in table 1.

**Table 1:** Selected sociodemographic characteristics of analytic sample

Characteristic	Total	
	n*	%
Age (years)		
16–19	656	41.30
20–24	932	58.70
Gender identity		
Woman (including trans woman)	880	55.40
Man (including trans man)	649	40.90
Identify gender in another way†	59	3.70
Identify as trans		
No	1511	95.80
Yes	53	3.40
Not sure	13	0.80
Prefer not to say	1	0.10
Sexual identity		
Heterosexual/straight	1021	64.50
Bisexual	264	16.70
Multiple sexual identities‡	90	5.70
Gay or lesbian	65	4.10
Pansexual	45	2.80
Asexual	33	2.10
Queer	22	1.40
Not sure	25	1.60
Prefer not to say	9	0.60
Prefer to self-describe	8	0.50
Ethnicity		
White Scottish or White British	1333	84.00
White, not Scottish or British	124	7.80
Asian, Asian Scottish or Asian British	54	3.40
Mixed or multiple ethnic groups	41	2.60
Black African, Caribbean, Black Scottish or Black British	20	1.30
Other ethnic group	10	0.60
Prefer not to say	4	0.30
Not sure	1	0.10
Disability, physical or mental health condition		
No	1170	73.80
Yes	327	20.60
Not sure	65	4.10
Prefer not to say	23	1.50
Main living arrangement in past year		
With my family/carers (eg, parent or guardian)	797	50.90
In a flat or house share	331	21.10
With my partner	200	12.80
In student accommodation	118	7.50
On my own	107	6.80
Experienced homelessness/no fixed accommodation	9	0.60
In supported accommodation	5	0.30
Scottish Index of Multiple Deprivation quintiles		
1 (most deprived)	252	19.30
2	238	18.20
3	230	17.60
4	260	19.90
5 (least deprived)	329	25.10

*Denominators vary across variables because of item non-response.

†Includes non-binary (n=38), prefer to self-describe (n=11) and prefer not to say (n=10).

‡Includes people who ticked multiple sexual identity categories (eg, bisexual AND queer; queer AND prefer to self-describe).

Approximately one-quarter of condom/contraception-using respondents reported that social distancing measures had made a difference to their access or use, while 59.8% indicated that social distancing measures had not made a difference, and 15.3% were unsure (table 2). Compared with men, greater proportions of women and those identifying their gender in another way reported that social distancing had impacted their access to, or use of, condoms and contraception (respectively 20.3% vs 27.8% and 32.2%).

**Table 2:** Perceived impact of social distancing measures on access to, or use of, condoms and contraception among young people who use condoms and contraception, by gender identity

Response	Have social distancing measures (ie, because of coronavirus) made any difference to the way you get or use condoms or contraception?							
	Gender identity							
	Women*		Men*		Identify in another way		Total	
	n	%	n	%	n	%	n	%
No	511	58.1	406	62.6	32	54.2	949	59.8
Yes	245	27.8	132	20.3	19	32.2	396	24.9
Not sure	124	14.1	111	17.1	8	13.6	243	15.3
Total	880	100.0	649	100.0	59	100.0	1588	100.0

*Women includes trans women; men includes trans men. Some 3.4% of the analytic sample identified as trans.

†Includes non-binary (n=38), prefer to self-describe (n=11) and prefer not to say (n=10).

### Analysis of open-text responses

Among the quarter of young people (n=396) who indicated that social distancing measures had made a difference to their access or use of condoms and contraception, 91% entered an open-text response, ranging from one word to detailed explanations (range: 1–181 words; mean: 21 words). Of the 361 submitted responses, 62.5% were by women, 33% by men and 4.5% by those identifying their gender in another way.

A small minority of responses indicated that social distancing measures had made a positive difference to the way they accessed or used condoms and contraception; for example, “*I can now get them* [unspecified] *sent to my house which makes me more comfortable*” [Man, 23, heterosexual/straight] and *“It has actually been easier which is a relief. I was able to email my GP practice for my prescription request and pick it up from the pharmacy without having to physically go to my practice or make an appointment”* [Woman, 22, heterosexual/straight].

A further minority described changes to access and use in neutral terms with no/little sentiment expressed, for example, “*Had to order condoms online*” [Man, 22, gay], “*Before I went to a sexual health clinic, now I get a prescription from the GP*” [Woman, 20, bisexual] and “*Started on a different contraception*” [Woman, 23, heterosexual/straight].

**Figure 1:**
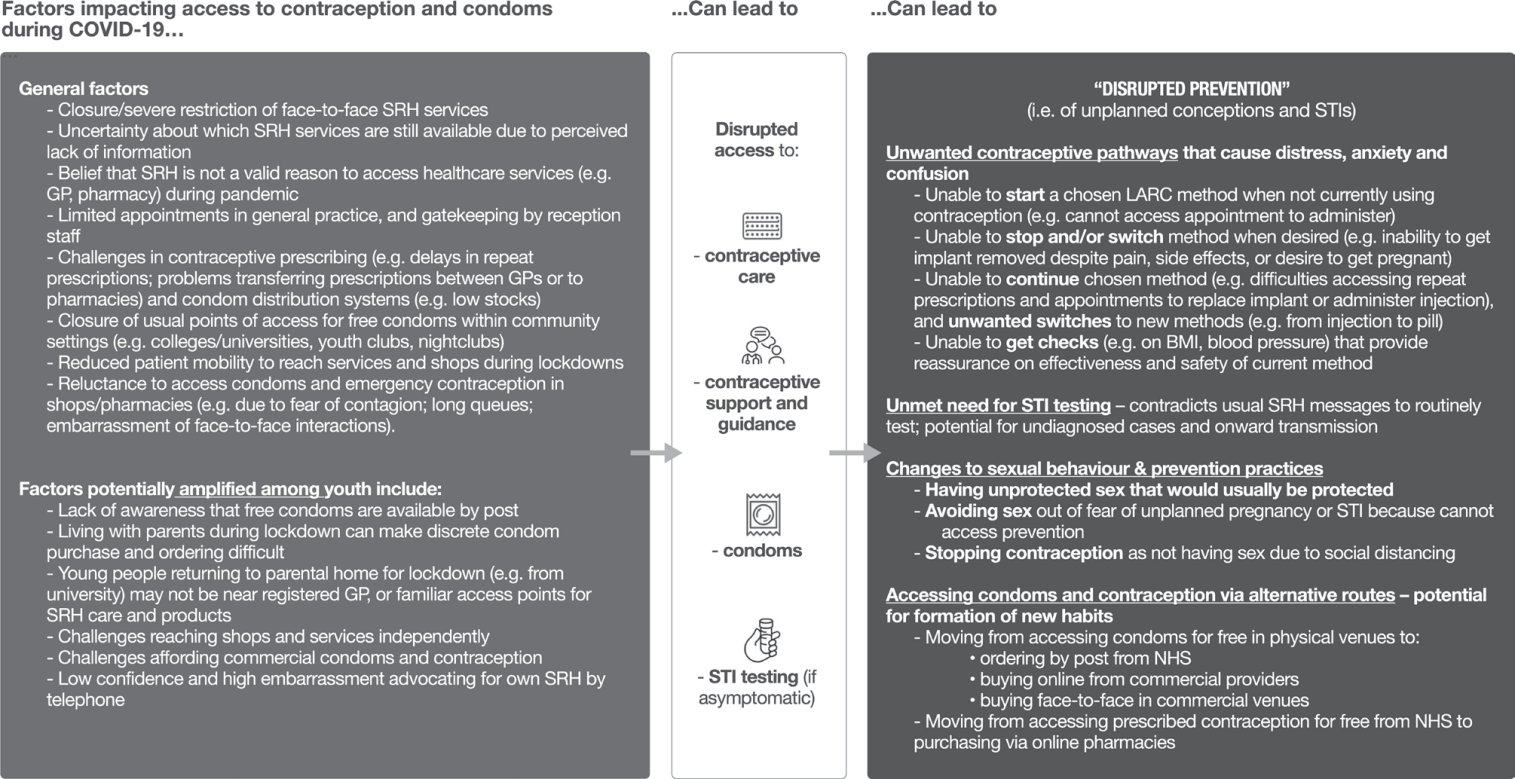
Thematic overview of young people’s open-text responses (n=361) explaining how social distancing measures have affected their condom and contraceptive access and use. BMI, body mass index; GP, general practitioner; LARC. long-acting reversible contraception; NHS, National Health Service; SRH, sexual and reproductive health; STI, sexually transmitted infection.

The vast majority of young people’s comments, however, indicated negative experiences and a plethora of challenges related to condom and contraceptive access and use. Figure 1 presents a thematic overview of young people’s accounts of factors affecting their access to condoms and contraception. Overall, the picture is one of *disrupted prevention*, including changes to sexual risk-taking and preventive practices, unwanted contraceptive pathways, unmet need for STI prevention, and alternative routes of access to condoms and contraception. Below, we build on these accounts to highlight three overarching barriers to young people’s pregnancy and STI prevention which may be less immediately apparent, but could potentially have longer-term consequences. Illustrative extracts relating to these three barriers are presented in table 3.

**Table 3:** Extracts from young people’s open-text responses (n=361) illustrating emerging barriers to pregnancy and sexually transmitted infection prevention in the context of COVID-19

Themes	Illustrative extracts
Self-censoring of SRH needs	*“Don’t want to bother the GP practice at this time for the contraceptive pill.”* [Woman, 19, heterosexual/straight] *“Don’t want to go to GP or pharmacy as I feel like I’m wasting time that they could help someone else.”* [Man, 16 heterosexual/straight] *“I have found it difficult as most places only have time to see you if it’s an emergency and I feel embarrassed prioritising getting contraception over someone who could have a serious problem.”* [Woman, 19, heterosexual/straight] *“Although it isn't, it seems 'unnecessary' compared to other things.”* [Woman, 20, bisexual] *“At a pharmacy you have to queue to get in and feel like condoms really aren’t that necessary so feel like you are wasting people’s time who really need to be there.”* [Woman, 21, heterosexual/straight] *“Don’t feel that it is appropriate to go in and use the c-card scheme at the pharmacy right now so buy my own.”* [Man, 17, gay]
Emerging confusion and anxiety about positive prevention in context of contradictory messages	**Confusion and anxiety about changes to contraception care** *“I have the implant which I was told lasted 3 years and it ran out a couple of months ago, now I’ve been told it lasts for 4 years? I’ve checked online and nothing backs this up. Very worrying.”* [Woman, 24, heterosexual/straight] *“I haven't had my blood pressure checked when I collected my prescription for the pill. Not sure when that will be possible again or the possible impact this will have.”* [Woman, 20, bisexual] *“Nurse ordered me another 6 month prescription of the pill over the phone without being checked despite being asked to go for a check up last time I got more of the pill.”* [Woman, 19, heterosexual/straight] *“I would usually go in to my GP to make sure everything is okay and if I was having any issues before getting a top up. I needed a new type of birth control at the start of lockdown as the one I was on caused a lot of bleeding. Instead of an appointment I was just given a new one to try without a discussion with a doctor.”* [Woman, 23, heterosexual/straight] *“It means I can’t get my blood pressure checked to make sure my contraception isn’t negatively impacting my health.”* [Woman, 20, heterosexual/straight] **Confusion and anxiety about changes to STI testing** *“I regularly attended or pre-booked appointments with a sexual health clinic before or after sexual encounters mainly for a chat, information or peace of mind testing but recently from Covid-19 I found this a difficult task or I felt that it would not be (or considered to be) essential.”* [Man, 22, bisexual] *“I've waited months past since I have had symptoms of an STI/D due to not wanting to break social distancing/not sure how sexual health clinics have been dealing with it.”* [Man, 21, pansexual] *“Currently I have been trying to get a routine sexual health screening before I have sex with new people however due to lockdown the sex clinic in my area is only taking emergency appointments.”* [Woman, 24, heterosexual/straight] *“I have been sexually active during the lockdown with multiple partners. It has been extremely disappointing that the sexual health clinics have (and still are) closed. I am unable to order STI postal kits. Why? I phone up the clinic and they said that they are not testing.”* [Woman, 24, heterosexual/straight] *“I have had to seek out STI screening from a private company as the NHS service I use told me that I needed symptoms of something before I could be screened. I did NOT have this problem pre-lockdown.”* [Woman, 24, bisexual]
Exacerbation of existing barriers to accessing condoms and contraceptive care	**Gatekeeping of services** *“It’s almost impossible to get an appointment to receive my birth control injection. Not only that but I have been frequently judged and even asked, ‘Do you really think that is important right now?’ when making an appointment.”* [Woman, 23, heterosexual/straight] *“Harder to get an appointment. Seems like only the most urgent people get seen. Puts people off fighting for an appointment.”* [Woman, 24, bisexual] **Challenges advocating for own SRH needs** *“Had to get blood pressure check before they would renew my pill. Eventually after having run out for a month I told my Mum who sorted it out for me.”* [Woman, 16, heterosexual/straight] *“Difficult to get in contact with GP to get contraceptive pill - could not go to GP for blood pressure check, etc. I was encouraged to change pill which I strongly did not want to do as it had previous adverse effects. After standing my ground, I got my prescription. However, when I was a few years younger I would have just left it and stopped taking my pill to avoid the difficult conversations.”* [Woman, 21, heterosexual/straight] **Low confidence interacting remotely with healthcare professionals** *“Due to the virus, I've needed to phone my doctors and talk on the phone however I struggle to talk on the phone. So it’s hard for me to order my contraception.”* [Prefer to self-describe gender, 18, pansexual] *“I’m too embarrassed to call up about it and I’m scared they will not allow me to have free condoms as I am a girl looking for them for me and my boyfriend.”* [Woman, 16, heterosexual/straight] *“Had a lot of issues trying to get the contraceptive pill whilst in lockdown. Had issues with it and hard to explain to male doctors or on telephone.”* [Woman, 18, bisexual] **Pandemic-related constraints on privacy and independence** *“Shops haven’t been opened resulting in not getting contraceptives and not being able to order them without keeping it a secret from family members.”* [Man, 16, heterosexual/straight] *“I no longer do my own shopping because I’m shielding, so I feel embarrassed asking my mum to buy condoms.”* [Woman, 19, queer] *“I used to just buy condoms at the shops but with Covid I had to move in with my parents and my dad was doing the shopping… bit awkward asking him to get his daughter some condoms.”* [Woman, 23, heterosexual/straight] **Changes to usual points of access that allow young people to overcome access barriers** *“Because the online service was temporarily stopped I didn’t know where to get contraception for free and couldn't afford it.”* [Man, 22, asexual] *“Haven't been able to get free condoms as I've been unaware whether sexual health clinics were open and my usual place of obtaining them* [my University] *was shut.”* [Man, 24, bisexual] *“I get my condoms from a youth group which is shut due to social distancing and do not feel comfortable getting them somewhere else.”* [Man, 16, pansexual] *“The only place I knew and felt comfortable with getting condoms from was public bathrooms.”* [Man, 18, bisexual] *“… the one-way system and social distancing at the supermarkets has made it more embarrassing to buy from the shelves!”* [Woman, 21, heterosexual/straight] *“Would normally get them late at night at supermarket but nowhere’s open and I’d be too embarrassed to get them anywhere or anytime else.”* [Man, 19, bisexual]

GP, general practitioner; NHS, National Health Service; SRH, sexual and reproductive health; STI/D, sexually transmitted infection/disease.

#### Emerging barriers to pregnancy and STI prevention in the context of COVID-19

##### Self-censoring of SRH needs

Although elements of SRH services were deemed essential to keep running during lockdown, public health messaging to only seek help for essential services and reduce pressure on general practice and pharmacy services appears to have been problematic as young people were unsure what counted as essential care, leading some to self-censor their need (eg, for contraceptive care). Consequences reported by some included discontinuing contraception, with potential implications for unintended conceptions and abortion.

##### Emerging confusion about positive prevention in the context of contradictory messages

The responses conveyed a strong sense of anxiety and confusion regarding sudden changes to standard care and routine advice about contraception and STI prevention. Where previous messaging had been absorbed about the importance of certain measures (eg, blood pressure and BMI checks for prescription of the pill, timely replacement of implant, asymptomatic STI testing), young people became confused because ‘new’ advice issued during lockdown (eg, no need for checks; that it is safe to extend use of implant beyond 3 years; that asymptomatic testing is not essential) contradicted this. Young people sought confirmation regarding this new advice online, but struggled to find trusted sources they could easily understand. In the absence of reassurance, confusion and anxiety appeared to take hold among some young people.

##### Exacerbation of existing barriers to accessing condoms and contraceptive care

Pandemic-related changes to provision of SRH products and services introduced new complexities within an already complex system. Young people’s accounts indicate how barriers to accessing condoms and contraception that existed pre-Covid (eg, embarrassment about face-to-face interactions to get condoms, discomfort directly interacting with healthcare professionals, challenges getting appointments) were exacerbated during the pandemic, and further compounded by a reduction in resources that previously enabled some to traverse these barriers (eg, closure of community-based access points and dedicated young people’s SRH services). Thus, obtaining SRH care during the pandemic required young people to advocate for their own SRH needs – a process inhibited by multiple factors, including gatekeeping of services (eg, by GP receptionists), lack of confidence interacting with healthcare professionals via telephone or video call, and constraints on independence and privacy to access SRH care if living in a parental home. Some received social support (eg, from parents, friends) to navigate these barriers; others did not. For some young people, these challenges led to discontinuing condom or contraception use altogether. While some sought to access products and STI testing via retail settings, including online shops and pharmacies, those who could not afford to went without, despite continuing to have sex.

## Discussion

Our study provides insights into young people’s disrupted access to, and use of, free condom and contraceptive services during the early stages of the COVID-19 pandemic. Evident in their accounts is considerable anxiety, uncertainty and frustration due to lack of clarity about the legitimacy of trying to access SRH services during a pandemic, contradictions to advice previously received, and intensified difficulties navigating what was already a complex system of care.

As discussions about recovery strategies for SRH begin, we suggest two ‘big picture’ questions that merit particular consideration. First, how do we best address confusion arising from changes to routine advice? Consensus about, and communication of, relative risks in a rapidly evolving pandemic is inevitably complex. However, without action to quickly clarify and widely communicate the rationale for changes in guidance, there is the possibility for these conflicting messages to sow seeds of confusion that undermine positive preventative practices for future generations coming of sexual age. It will be important to collaborate with young people to create messages that are easily understood and accessed, including via digital media.^19^ Clarity in messages would also support understanding among young people that SRH services are, in fact, essential primary healthcare services.^8 15^

Second, how do we mitigate against potential widening of SRH inequalities opened up by uneven access to the resources (eg, material, social support, transport, digital access and health literacy) to navigate complex and rapidly changing SRH systems and information? Telemedicine, for instance, may offer new tools for contraceptive consultations,^14 20^ but is unlikely to be a panacea to provision for young people who lack the confidence or social support to communicate their needs to a healthcare professional remotely. While switching to paying for condoms and contraception may offer an easy and convenient alternative for some, financial barriers prohibit these routes for others. Moreover, a potential unintended consequence of allowing commercial markets to displace NHS-run condom schemes and contraceptive services is the loss of opportunities for health promotion messaging and monitoring of contraceptive use within the context of young people’s wider health and well-being.

Strengths of this study include an open-text question, co-designed with young people, and allowing respondents to express issues of greatest salience to them, using their own words. Limitations include the study's cross-sectional design, precluding assessment of change over time. While use of a convenience sample limits generalisability, our data reflect experiences of population groups who face considerable barriers to SRH care (eg, gender, sexual and ethnic minorities; young people with disabilities).

## Recommendations and conclusions

Professional bodies have developed recommendations and guiding principles for restoration of SRH services in the ongoing COVID-19 pandemic.^21 22^ Below, we outline additional suggestions to support continuity of free condom and contraceptive services to young people.

- Emphasis on public SRH messaging that supports young people to easily navigate SRH care within the context of evolving restrictions. Messaging should be clear, co-produced with young people, and communicated widely via digital and social media platforms. Messaging should convey: 1) that access to SRH services is a fundamental right, and provision of contraception is essential care; 2) that changes in standard SRH care (eg, relaxation of requirements for blood pressure and BMI checks, extended use of certain forms of LARC) are safe but not best practice, which will be returned to post-pandemic.
- Prioritisation of structures that enable cross-sector sharing of resources and learning (eg, between national and local government, NHS, third sector) to enable the SRH workforce to quickly develop public messaging that is consistent, responsive to changing restrictions, and effectively communicated.
- Greater investment in improving digital sexual health literacy among young people, including how to find and evaluate accurate information online.
- Wider promotion of existing digital tools that empower young people’s contraceptive decision-making and use (eg, the Contraceptive Choices website).
- Prioritisation of choice in SRH consultation mode (eg, telephone, text messaging, video, in-person) so that young people can access help confidentially using forms of communication with which they feel most comfortable.

In conclusion, positive action (underpinned by adequate resources) will be required to ensure that emerging barriers to STI and pregnancy prevention within the context of COVID-19 do not undermine positive prevention practices, and widen SRH inequalities among young people in the longer term.
